# Refining Alzheimer's disease biological diagnosis with plasma biomarkers: Resolving p‐tau217 “gray zone” with p‐tau181 integration

**DOI:** 10.1002/dad2.70285

**Published:** 2026-02-15

**Authors:** Giulia Giacomucci, Silvia Maria Rita Tabbì, Assunta Ingannato, Silvia Bagnoli, Sonia Padiglioni, Chiara Crucitti, Chiara Sensi, Serena Sanesi, Valentina Moschini, Carmen Morinelli, Giulia Galdo, Valentina Berti, Benedetta Nacmias, Valentina Bessi

**Affiliations:** ^1^ Department of Neuroscience, Psychology, Drug Research and Child Health University of Florence Florence Italy; ^2^ IRCCS Fondazione Don Carlo Gnocchi Florence Italy; ^3^ Regional Referral Centre for Relational Criticalities ‐ Tuscany Region Florence Italy; ^4^ University of Florence Florence Italy; ^5^ Neurology Unit, Dipartimento Neuromuscoloscheletrico E Degli Organi Di Senso Careggi University Hospital Florence Italy; ^6^ Department of Biomedical, Experimental and Clinical Sciences “Mario Serio” University of Florence Florence Italy; ^7^ Nuclear Medicine Unit Azienda Ospedaliero‐Universitaria Careggi Florence Italy

**Keywords:** Alzheimer's disease, mild cognitive impairment, plasma biomarkers, plasma GFAP, plasma NfL, plasma p‐tau181, plasma p‐tau217, subjective cognitive decline

## Abstract

**BACKGROUND:**

Blood‐based biomarkers offer a less invasive and more scalable alternative to cerebrospinal fluid (CSF) analysis and amyloid‐positron emission tomography (PET) for the biological diagnosis of Alzheimer's disease (AD). Among blood‐based biomarkers (BBMs), plasma phosphorylated tau217 (p‐tau217) has shown the highest accuracy, although intermediate (“gray zone”) values remain challenging to interpret.

**METHODS:**

In this study, 401 individuals across the Alzheimer's Disease (AD) continuum (Subjective Cognitive Decline, Mild Cognitive Impairment, and AD dementia) underwent clinical and biomarker assessment. Plasma p‐tau217, p‐tau181, neurofilament light chain (NfL), and glial fibrillary acidic protein (GFAP) were measured. Core1 status was defined through CSF or amyloid‐PET.

**RESULTS:**

Plasma p‐tau217 demonstrated the strongest discrimination of Core1 positivity (area under the curve [AUC] = 0.95) and showed the steepest increase with disease progression. A two‐cutoff strategy improved diagnostic accuracy (94%), though 18% of patients fell into the gray zone. Within this subgroup, p‐tau181 was the only predictor of Core1 status and correctly reclassified 77.4% of indeterminate cases.

**DISCUSSION:**

These findings support a sequential plasma biomarkers approach for reliable AD detection.

## Highlights

- Plasma phosphorylated tau217 (p‐tau217) showed the highest accuracy for detecting Alzheimer's disease (AD) pathology.
- A two‐cutoff strategy for p‐tau217 improved diagnostic reliability.
- About 18% of patients displayed intermediate p‐tau217 “gray zone” values.
- p‐tau181 was the only biomarker that resolved p‐tau217 gray zone cases.
- Sequential use of p‐tau217 and p‐tau181 enhanced classification accuracy.

## 1 BACKGROUND

Blood‐based biomarkers (BBMs) have recently emerged as a major advancement in the diagnosis of Alzheimer's Disease (AD).^1^ Traditionally, in vivo identification of AD pathology relied on cerebrospinal fluid (CSF) analysis and amyloid positron emission tomography (amyloid‐PET), which detect amyloid‐β (Aβ) deposition and tau pathology.^2^, ^3^ While these modalities remain the assays for biological diagnosis of AD, their limited accessibility, invasiveness, and high cost have restricted their implementation in routine clinical practice and large‐scale screening programs. The development of highly sensitive immunoassay technologies has enabled the reliable quantification of key AD‐related proteins in plasma, which have been recently included in the Alzheimer's Association Revised Criteria for AD diagnosis.^4^ Among these, phosphorylated tau217 (p‐tau217) and p‐tau181 reflect Aβ‐driven tau phosphorylation, neurofilament light chain (NfL) indicates neuroaxonal injury, and glial fibrillary acidic protein (GFAP) reflects astroglial activation.^5^, ^6^, ^7^ Collectively, these biomarkers capture complementary aspects of AD pathophysiology, promoting a shift toward a less invasive, scalable, and biologically grounded diagnostic approach.

Recent evidence consistently demonstrated that plasma p‐tau217 outperforms other BBMs in identifying AD pathology, achieving an extremely high accuracy even in the earliest disease stages.^8^, ^9^, ^10^, ^11^ To further enhance diagnostic performance, a two‐cutoff approach—one maximizing sensitivity and one maximizing specificity—has been proposed, meeting the minimal diagnostic standards recommended by the Global CEO Initiative on AD.^12^ However, a proportion of individuals still present intermediate or “gray zone” p‐tau217 concentrations, thus refining classification in these indeterminate cases remains a key challenge.^9^, ^13^ In this context, we hypothesized that combining multiple plasma biomarkers may improve diagnostic precision by leveraging distinct yet complementary biological signals. In this study, we aimed to: (i) characterize the trajectories of plasma p‐tau217, p‐tau181, NfL, and glial fibrillary acidic protein (GFAP) across the AD continuum—from Subjective Cognitive Decline (SCD) to Mild Cognitive Impairment (MCI) and AD dementia; (ii) establish optimal cutoff values for each biomarker in predicting AD pathology; (iii) evaluate whether incorporating additional plasma biomarkers could improve classification accuracy in patients with indeterminate p‐tau217 levels; (iv) propose a flow chart for the use and the interpretation of plasma biomarkers to detect AD since the earliest stages of cognitive decline.

## 2 MATERIALS AND METHODS

### 2.1 Participants

We enrolled 401 patients (77 SCD, 188 MCI and 136 AD demented) referring to the Centre for Alzheimer's Disease and Adult Cognitive Disorders of Careggi Hospital in Florence between July 2018 and May 2025.

Patients met the following inclusion criteria:

1. receiving a clinical diagnosis of AD dementia according to the National Institute on Aging—Alzheimer's Association (NIA‐AA) criteria, including the atypical variants^14^,
2. receiving a clinical diagnosis of MCI according to NIA‐AA criteria^15^,
3. receiving a clinical diagnosis of SCD according to SCD‐I criteria^16^.

At baseline, patients underwent comprehensive family and clinical history, neurological examination and extensive neuropsychological investigation (described in detail elsewhere,^17^) blood collection for measurement of plasma p‐tau217, p‐tau181, NfL and GFAP concentration and genetic analysis. Plasma p‐tau217 were measured using fully automated CLEIA on the LUMIPULSE G600II system according to the manufacturer guidelines (Lumipulse® assay, Fujirebio), while p‐tau181, NfL, and GFAP analyses were performed on the automated single molecule assay (Simoa) SR‐X platform (Quanterix corp.).

Age at onset was defined as the age at which the patient first began experiencing cognitive symptoms. A positive family history of dementia was defined as having one or more first‐degree relatives with a documented history of cognitive decline. Apolipoprotein E (*APOE)* genotyping was available for 386 patients. Patients underwent assessment of AD biomarkers: CSF biomarker analysis was performed as the first‐line approach, while amyloid‐PET was used in those who refused lumbar puncture. In particular, 370 patients (64 SCD, 177 MCI, 129 AD‐d) underwent CSF collection for Aβ42, Aβ42/Aβ40, t‐tau, p‐tau, and p‐tau/Aβ42. One hundred and ten patients (40 SCD, 45 MCI and 25 AD‐d) underwent amyloid‐PET scans. Both CSF collection and amyloid‐PET scans were performed in 79 patients (26 SCD, 34 MCI and 19 AD‐d). Methods for CSF collection and analysis, plasma biomarkers’ analyses, and amyloid‐PET acquisition are described in Supplementary Materials.

**RESEARCH IN CONTEXT**

- **Systematic review**:  Previous studies consistently showed plasma phosphorylated tau217 (p‐tau217) as the most accurate blood‐based biomarkers (BBMs) for detecting Alzheimer's disease (AD) pathology, with two‐cutoff strategies proposed to improve clinical interpretability. However, a substantial proportion of individuals show p‐tau217 values in an intermediate “gray zone,” and there is limited evidence on how to classify such cases.
- **Interpretation**: In a real‐world cohort across the AD continuum, we confirmed p‐tau217 as the strongest predictor of underlying AD pathology, achieving excellent diagnostic accuracy. Importantly, p‐tau181 was the only biomarker that improved classification in individuals with gray‐zone p‐tau217 levels, enabling correct reclassification in most cases.
- **Future directions**: Future studies should validate this sequential approach in independent and more diverse populations, determine optimal retesting intervals, and evaluate its impact on clinical decision‐making and treatment eligibility.

### 2.2 Classification of patients according to the revised criteria of Alzheimer's Association Workgroup

Based on biomarker results, patients were classified according to the Revised Criteria of Alzheimer's Association Workgroup. Patients were rated as Core1+ in case of abnormality on at least one of specific Core1 biomarkers (amyloid PET, CSF Aβ42/Aβ40, CSF p‐tau 181/Aβ42), and as Core1− in case of normal Core1 biomarkers.^4^ Patients were furtherly classified according both to diagnosis and Core1 biomarkers’ results as follows: SCD Core1−, SCD Core1+, MCI Core1−, MCI Core1+, AD‐d (all Core1+) (Supplementary Table 1 and Supplementary Table 2).

### 2.3 Statistical analysis

Statistical analyses were performed using R 4.5.1 (R Foundation for Statistical Computing, Vienna). All tests were two‐tailed with a significance threshold of *p *< 0.05. Continuous variables were summarized as means ± standard deviation, and categorical variables as frequencies or percentages with 95% confidence intervals (CIs). Group comparisons were conducted using chi‐squared tests for categorical data and one‐way analysis of variance (ANOVA) followed by Tukey post‐hoc tests for continuous variables. Effect sizes were estimated with partial η^2^, Cohen's d, rank biserial correlation (r), and Cramer's V. Linear models were used to adjust for potential confounders, and logistic regression to identify variables associated with Core1 positivity (i.e., underlying AD pathology). LOESS (locally estimated scatterplot smoothing) regression analyses were performed to model trajectories of plasma biomarkers (p‐tau217, p‐tau181, NfL, GFAP) across the AD continuum, after z‐score standardization based on the SCD Core1− group. Receiver operating characteristic (ROC) analyses were performed to assess each biomarker's ability to discriminate Core1+ from Core1− patients, calculating the corresponding areas under the curve (AUCs). DeLong's test was used to compare AUCs across biomarkers. A combined model including all biomarkers was also constructed using logistic regression analysis. The AUC of this combined model was compared to those of the individual biomarkers to determine whether it provided a better overall performance in distinguishing Core1+ from Core1− patients. For p‐tau181, NfL, and GFAP, a single optimal cutoff was defined by maximizing the sum of sensitivity and specificity, whereas for p‐tau217 a two‐cutoff approach was applied: the lower achieving 95% sensitivity and the upper achieving 95% specificity. Based on the calculated cutoff values, patients were categorized as positive or negative for p‐tau181, NfL, and GFAP. Regarding p‐tau217, patients were classified into three groups using two different thresholds: patients with p‐tau217 values above the upper cutoff were considered positive, those with p‐tau217 values below the lower cutoff were considered negative, while those with p‐tau217 values falling between the two cutoffs were classified as belonging to the gray zone, as previously described.^18^ Diagnostic accuracy was expressed through standard metrics (accuracy, sensitivity, specificity, positive predictive value [PPV], negative predictive value [NPV]), with 95% CIs estimated by bootstrap resampling (1000 iterations).

To further classify patients with gray‐zone p‐tau217 levels, a generalized linear model (GLM) was applied to explore which plasma biomarkers (p‐tau181, NfL, GFAP; positive/negative according to respective cutoffs) were associated with Core1 positivity. The GLM‐identified biomarker was then applied to reclassify gray‐zone patients, and performance of the combined strategy was compared with p‐tau217 alone using bootstrap testing of accuracy differences and McNemar's test for paired outcomes.

## 3 RESULTS

### 3.1 Comparison of plasma biomarkers levels among diagnostic and Core1 subgroups

All plasma biomarkers significantly differed among groups, even after adjusting for age and sex (p‐tau217 F [6229] = 17.20, *p *< 0.001, adj. *R* ^2 ^= 0.290; p‐tau181 F [6,237] = 24.61, *p *< 0.001, adjusted *R* ^2^ = 0.368; NfL F [6,356] = 15.82, *p *< 0.001, adjusted *R* ^2^ = 0.196; F [6248] = 14.15, *p *< 0.001, adjusted *R* ^2^ = 0.237) (Table 1, Supplementary Figure 1).

**TABLE 1:** Comparison of plasma biomarkers levels in patients with SCD, MCI, and AD‐d classified according to the Alzheimer's Association Revised Criteria for Alzheimer's disease diagnosis.

Parameter	SCD Core1−(*N* = 54)	SCD Core1 + (*N* = 23)	MCI Core1−(*N* = 94)	MCI Core1 + (*N* = 94)	AD‐d (*N* = 136)
Age at plasma collection (years)	63.00 ± 7.70^a^, ^b^, ^c^, ^d^	68.00 ± 9.20^a^	68.00 ± 8.90 ^b^, ^g^	73.00 ± 7.00 ^c^, ^g^	71.00 ± 6.30 ^d^
Sex (F – M)	40 – 14 ^b^, ^c^, ^d^	13 – 10	53 – 41 ^b^	53 – 41 ^c^	79 – 57 ^d^
*APOE* ε4	27.80% ^c^, ^d^	34.80%	19.10% ^g^, ^h^	56.40% ^c^, ^g^	51.50% ^d^, ^h^
Plasma p‐tau217 (pg/ml)	0.19 ± 0.14 ^a^, ^c^, ^d^	0.65 ± 0.5 ^a^, ^e^, ^f^	0.18 ± 0.09 ^e^, ^g^, ^h^	0.74 ± 0.52 ^c^, ^g^, ^i^	1.60 ± 1.60 ^d^, ^f^, ^h^, ^i^
Plasma p‐tau181 (pg/ml)	1.90 ± 0.81 ^a^, ^c^, ^d^	2.90 ± 0.79 ^a^, ^f^	2.10 ± 0.95 ^g^, ^h^	3.80 ± 1.50 ^c^	4.40 ± 1.80 ^d^, ^f^, ^g^, ^h^
Plasma NfL (pg/ml)	12.00 ± 5.70 ^a^, ^c^, ^d^	17.00 ± 9.20 ^a^	15.00 ± 6.30 ^g^, ^h^	21.00 ± 13.00 ^c^	23.00 ± 10.00 ^d^, ^g^, ^h^
Plasma GFAP (pg/ml)	160.00 ± 67.00 ^c^, ^d^	260.00 ± 180.00	210.00 ± 120.00 ^g^, ^h^	310.00 ± 140.00 ^c^, ^g^	360.00 ± 200.00 ^d^, ^h^

*Note*: Values are reported as mean and standard deviation. Statistical significance is set at *p *< 0.05. Statistically significantly different values between the groups are reported as underlined character and refer to:

Abbreviations: AD‐d, Alzheimer's disease dementia; APOE, apolipoprotein E; GFAP, glial fibrillary acidic protein; MCI, mild cognitive impairment; NfL, neurofilament light chain; p‐tau, phosphorylated tau; SCD, subjective cognitive decline.

^a^ SCD Core1− vs SCD Core1+

^b^ SCD Core1− vs MCI Core1−

^c^ SCD Core1− vs MCI Core1+

^d^ SCD Core1− vs AD‐d

^e^ SCD Core1+ vs MCI Core1−

^f^ SCD Core1+ vs AD‐d

^g^ MCI Core1− vs MCI Core1+

^h^ MCI Core1− vs AD‐d

^i^ MCI Core1+ vs AD‐d.

Considering plasma p‐tau217, AD‐d patients showed the highest concentrations. Among MCI patients, Core1+ individuals had higher plasma p‐tau217 levels than Core 1‐ (*p *< 0.001); plasma p‐tau217 levels were higher in SCD Core1+ than in SCD Core1− (*p *< 0.001) and in MCI Core1− (*p *< 0.001). No significant differences were observed between MCI Core1+ and SCD Core1+ (*p *= 0.984). Similarly, AD‐d patients exhibited the highest p‐tau181 levels. Within both MCI and SCD subgroups, Core1+ individuals had higher p‐tau181 concentrations than Core1− patients (*p *< 0.001). As regard plasma NfL, the highest concentrations were observed in AD‐d patients, while MCI and SCD Core1+ participants had higher NfL levels than their Core1− counterparts. Interestingly, no significant differences were detected between SCD Core1+, MCI Core1+, and AD‐d groups. Finally, the highest GFAP levels were found in AD‐d patients, with significant differences compared to MCI Core1− (*p *< 0.001) and SCD Core1− (*p *< 0.001). MCI Core1+ patients showed higher GFAP levels than MCI Core1− (*p *= 0.001), while no significant differences were observed between SCD Core1+ and SCD Core1− (*p *= 0.079).

**FIGURE 1:**
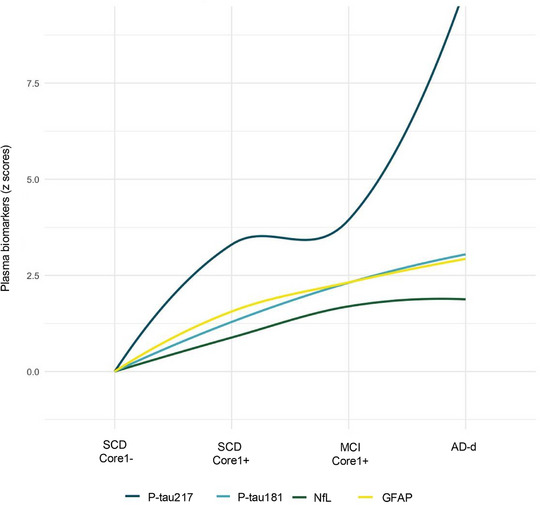
Locally estimated scatterplot smoothing (LOESS) trajectories of plasma biomarkers across the Alzheimer's disease (AD) continuum. Plasma concentrations of phosphorylated tau217 (p‐tau217), p‐tau181, glial fibrillary acidic protein (GFAP), and neurofilament light chain (NfL) were transformed into z‐scores using the subjective cognitive decline (SCD) Core1− group as reference. LOESS regression curves illustrate biomarker dynamics across clinical stages (SCD Core1−, SCD Core1+, mild cognitive impairment (MCI) Core1+ and AD dementia).

Locally estimated scatterplot smoothing (LOESS) curves were constructed to evaluate the trajectories of plasma biomarkers across the AD continuum (including SCD Core1−, SCD Core1+, MCI Core1+ and AD‐d). All plasma biomarkers increased progressively with advancing disease stage, but with distinct patterns. In more details, plasma p‐tau217 exhibited the steepest and most pronounced rise, with elevations already detectable in SCD Core1+, followed by a stabilization between SCD Core1+ and MCI Core1+, and a marked acceleration in AD dementia. Plasma p‐tau181 and GFAP demonstrated parallel, continuous increases with more moderate slopes, indicating a gradual but consistent association with disease progression. Finally, plasma NfL displayed the most attenuated changes, with only modest increases and a plateau in later stages (Figure 1).

### 3.2 Plasma biomarkers accuracies in predicting AD pathology and cutoffs definition

A logistic regression analysis was performed to determine which factors might influence the risk of presenting an underlying AD pathology, considering as covariates plasma p‐tau217, p‐tau181, NfL and GFAP levels, age at plasma collection, clinical diagnosis, sex and *APOE* genotype. The regression model was statistically significant (χ^2^ 475.24, *p *< 0.001). The model explained 99.03% (Nagelkerke *R* ^2^) of the variance. Among the covariates, only plasma p‐tau217 levels (B 9.16, OR = 9531.73, *p *< 0.001) were significant predictor of Core 1 status (i.e., presence or absence of an underlying AD pathology (Supplementary Table 3).

**FIGURE 2:**
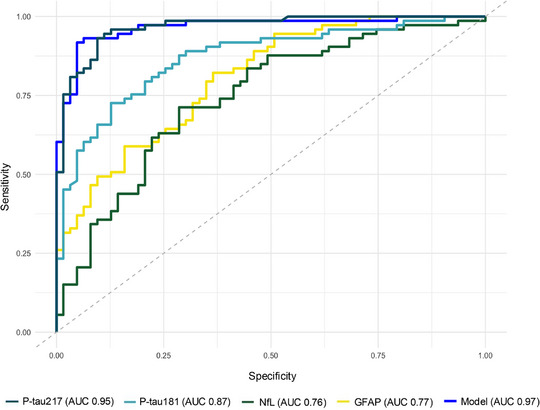
Receiver operating characteristic (ROC) curves of plasma biomarkers in predicting Alzheimer's disease (AD) pathology. Areas under the curve (AUCs) of individual plasma biomarkers (phosphorylated tau217 [p‐tau217], p‐tau181, neurofilament light chain [NfL], and glial fibrillary acidic protein [GFAP]) and of a combined model including all biomarkers was assessed for discriminating Core1+ from Core1− patients.

Then, ROC curve analyses showed that plasma p‐tau217 presented the highest AUC (0.95, 95%CI 0.93–0.98) than other biomarkers, as demonstrated with DeLong's test (Table 2). Using logistic regression analysis, a combined model including all plasma biomarkers was developed to predict Core1 status. The combined model demonstrated excellent diagnostic performance with an AUC of 0.97 (95%CI 0.94–0.99), but this was not superior to the AUC of plasma p‐tau217 alone (DeLong's test *p *= 0.50) (Figure 2). Optimal cutoff values for each plasma biomarker were developed by maximizing the Youden index, which identifies the threshold that maximizes the sum of sensitivity and specificity, providing the best overall classification accuracy.

- For plasma p‐tau181, the cutoff value was 2.23 pg/ml, identifying Core1+ patients with good accuracy (82.72% [95% CI 77.96–87.47]), sensitivity (91.03% [95% CI 87.47–96.84]), specificity (70.41% [95% CI 64.67–76.15], PPV 81.99% ([95% CI 77.16–86.82]) and NPV (84.15% [95% CI 79.55–88.74]).
- For plasma NfL, the cutoff value was 16.53 pg/ml, discriminating Core1+ from Core1− patients with fair accuracy (70.41% [95% CI 65.73–75.09]), sensitivity (67.11% [95% CI 62.29–71.93]), specificity (75.91% [95% CI 71.53–80.30], PPV 82.26% ([95% CI 78.34–86.18]), and a poor NPV (58.10% [95% CI 53.04–63.16]).
- For plasma GFAP, the optimal cutoff value was 249.98 pg/ml, identifying Core1+ patients with fair accuracy (69.92% [95% CI 64.30–75.54]), sensitivity (61.44% [95% CI 55.48–67.40]), good specificity (82.52% [95% CI 77.88–87.18], good PPV 83.93% ([95% CI 79.43–88.43]), and a poor NPV (59.03% [95% CI 53.00–65.05]).

**TABLE 2:** Comparison of AUC of plasma p‐tau217, p‐tau181, NfL, and GFAP in in predicting Core 1 status.

Parameter	AUC	95% CI	*p* vs p‐tau217
Plasma p‐tau217	0.95	0.93–0.98	—
Plasma p‐tau181	0.87	0.83–0.92	0.003
Plasma NfL	0.76	0.71–0.81	< 0.001
Plasma GFAP	0.77	0.71–0.82	< 0.001

*Note*: The table shows the AUC values with their 95% confidence intervals (CIs) and the *p* values from DeLong's test comparing each biomarker with p‐tau217.

Abbreivations: AUC, area under the curve; CI, confidence interval; GFAP, glial fibrillary acidic protein; NfL, neurofilament light chain; p‐tau, phosphorylated tau.

**FIGURE 3:**
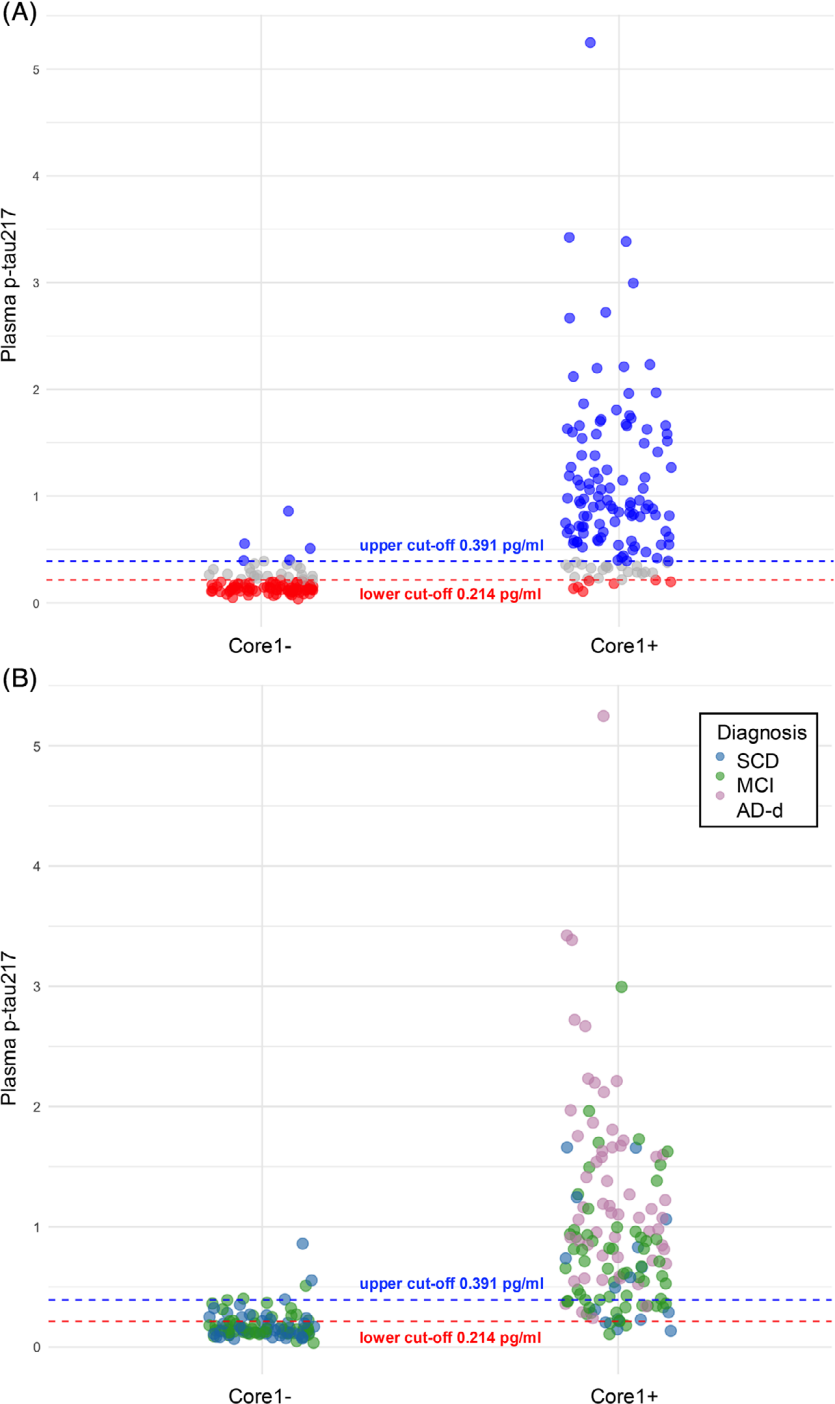
Two‐cutoff approach in discriminating Core1+ from Core1− patients in subjective cognitive decline (SCD), mild cognitive impairment (MCI), and Alzheimer's disease dementia (AD‐d). (A) Dot plot illustrating distribution of patients based of plasma phosphorylated tau217 (p‐tau217) levels categorization according to the two‐cutoff approach with sensitivity and specificity fixed at 95%. Blue dots: patients with plasma p‐tau217 levels above the upper cutoff (clearly positive). Red dots: patients with plasma p‐tau217 levels below the lower cutoff (clearly negative). Gray dots: patients with intermediate values of plasma p‐tau217 (gray zone). (B) Dot plot illustrating distribution of patients based on plasma p‐tau217 levels categorization according to the two‐cutoff approach with sensitivity and specificity fixed at 95%. Blue dots: SCD patients. Green dots: MCI patients. Pink dots: AD‐d patients.

Since plasma p‐tau217 proved to be the plasma biomarker with the highest AUC, thus representing the potential diagnostic plasma biomarker for AD, we applied the two cutoffs approach: the lower cutoff was set to achieve 95% sensitivity, and the upper cutoff was set to achieve 95% specificity. The upper cutoff (specificity of 95%) was 0.391 pg/ml, and the lower cutoff (sensitivity of 95%) was 0.214 pg/ml. Using the two‐cutoffs approach, plasma p‐tau217 had an overall excellent accuracy of 94.33% (95% CI 91.08–97.58), a PPV of 95.58% (95% CI 92.68–98.47), and an NPV of 92.59% (95% CI 89.91–96.28%) in predicting Core1 status. The grey zone group included 18.14% of patients (14 SCD, 25 MCI, and 4 AD‐d): 51.16% of them were Core1+ (Figure 3A).

Subsequently, distribution of patients based on ptau217 values was explored, considering the clinical diagnoses of SCD, MCI, and AD‐d (Figure 3B).

- In SCD subgroup, 58.06% of patients showed p‐tau217 levels below the lower cutoff, 19.35% above the upper cutoff, and 22.58% fell within the gray zone.
- In MCI subgroup, 38.79% of patients showed p‐tau217 levels below the lower cutoff, 39.65% above the upper cutoff, and 21.55% fell within the gray zone.
- In AD‐d subgroup, 93.22% of patients showed plasma p‐tau217 levels above the upper cutoff, while 6.77% fell within the gray zone.

### 3.3 Resolving p‐tau217 gray zone with other plasma biomarkers

To investigate whether additional plasma biomarkers could help resolve p‐tau217 gray zone and correctly classify patients as Core1+ or Core1−, we conducted a GLM including plasma p‐tau181, NfL, GFAP (as dichotomic variables, i.e. positive or negative based on previously established cutoffs), age, and sex. Only plasma p‐tau181 positivity was significantly associated with Core1 positivity. Specifically, individuals with positive p‐tau181 had markedly higher OR of Core1 positivity compared to those with negative p‐tau181 (OR 12.2; 95% CI 1.39–214.86; *p *= 0.040). None of the other predictors—including NfL, GFAP, age, or sex—demonstrated a statistically significant association with the outcome. Subsequent model selection based on the corrected Akaike Information Criterion (AICc) supported a parsimonious model including p‐tau181 as the sole predictor (AICc = 31.7, model weight = 0.44). Models incorporating additional biomarkers or demographic covariates did not substantially improve model fit (ΔAICc > 2) (Table 3, Supplementary Figure 2).

**TABLE 3:** GLM predicting Core1 positivity in p‐tau217 gray zone.

Predictors	B	OR	95% CI (Lower–Upper)	*p‐*value
Plasma p‐tau181 (positive vs negative)	2.503	12.22	1.39–214.86	**0.040**
Plasma NfL (positive vs negative)	−0.938	0.39	0.03–3.12	0.397
Plasma GFAP (positive vs Negative)	−0.463	0.63	0.04–7.17	0.716
Age (per year increase)	0.051	1.05	0.89–1.25	0.539
Sex (male vs female)	−0.299	0.74	0.07–7.29	0.795

*Note*: Results from a multivariable logistic regression model including plasma p‐tau181, NfL, GFAP, age, and sex. B refers to the log‐odds coefficient. Significant associations (*p *< 0.05) are reported in **bold character**.

Abbreviations: CI, confidence interval; GFAP, glial fibrillary acidic protein; GLM, generalized linear model; NfL, neurofilament light chain; OR, odds ration; p‐tau, phosphorylated tau.

Given the results of the model and the potential role of plasma p‐tau181 in clarifying the p‐tau217 gray zone, we aimed to evaluate whether including p‐tau181 could indeed improve patient classification. For this purpose, we considered patients with p‐tau217 levels within the gray zone: plasma p‐tau181 measurements were available for 31 of them. Patients within the gray zone were then classified as p‐tau181 positive or negative (according to the previously defined cutoff), in order to assess whether this could improve the classification of patients into Core1+ or Core1−.

Adding plasma p‐tau181 in patients from p‐tau217 gray zone allowed classification of these otherwise indeterminate cases. Of these, 24/31 (77.40%) were correctly classified and 7/31 (22.60%) misclassified (2/31 were false negative cases, while 5/31 were false positive ones). Then, we evaluated the global performance of this combined strategy (plasma p‐tau217 + p‐tau181). The combined strategy showed an excellent accuracy of 92.00% [95% CI 88.46–95.54], a PPV of 92.25% [95% CI 88.74–95.76], and a NPV of 91.67% [95% CI 88.06–95.28]. The difference in overall accuracy between p‐tau217 alone and the combined approach was not statistically significant (ΔAcc = −2.3%, 95% CI: −7.2 to +2.6, bootstrap *p *= 0.336) (Supplementary Table 4). Importantly, in the subgroup of p‐tau217 gray zone patients, the improvement achieved by adding plasma p‐tau181 was statistically significant (McNemar test, *p *< 0.001), demonstrating that plasma p‐tau181 correctly classified 77.40% of patients who were unclassifiable using plasma p‐tau217 alone (Supplementary Figure 3).

## 4 DISCUSSION

In this study, we investigated the diagnostic performance of multiple plasma biomarkers along the AD continuum, providing novel insights into their trajectories and clinical utility, focusing on p‐tau217 and its integration with other markers in detecting AD pathology in real world populations.

First, our results demonstrated that plasma biomarkers follow a distinct trajectory across disease stages. LOESS curve modeling revealed that plasma p‐tau217 exhibits the steepest increase when transitioning from cognitively unimpaired individuals without AD pathology to the first symptomatic stage of the AD continuum (Stage 2, namely, SCD with underlying AD). Interestingly, p‐tau217 levels appeared to stabilize between SCD due to AD (stage 2) and MCI (stage 3), suggesting that these two stages may be biologically similar.^13^ The most pronounced rise occurred at the transition from MCI to AD dementia. Conversely, p‐tau181 and GFAP showed a more gradual increase, less abrupt than that observed for p‐tau217, whereas NfL displayed a flatter incremental trend.

Consistent with recent literature, plasma p‐tau217 confirmed its position as the strongest predictor of underlying AD pathology, with an extremely high odds ratio.^9^, ^11^ Diagnostic accuracy analyses further demonstrated that p‐tau217 achieved an AUC of 0.95, significantly higher than those obtained by the other plasma biomarkers considered, and comparable to that of a combined model including all biomarkers. These results confirm the absolute superiority of p‐tau217 as a diagnostic biomarker for AD, showing excellent performance even when used alone.^8^, ^9^, ^11^, ^19^

In our cohort, specific cutoff values were identified for each biomarker to distinguish between AD and non‐AD patients. For p‐tau181, NfL, and GFAP, a single cutoff approach was used, yielding results in agreement with previous studies for p‐tau181^20^, ^21^ and GFAP,^22^ while NfL displayed slightly different thresholds—possibly reflecting population‐specific characteristics.^23^ Among these, p‐tau181 achieved the best accuracy, PPV, and NPV, as expected from a biomarker directly reflecting AD‐specific tau pathology, whereas NfL and GFAP, being less specific, showed lower discriminative power.^4^, ^24^

Given the outstanding discriminative capacity of p‐tau217 and its role as a future diagnostic plasma biomarker for AD,^4^, ^9^, ^10^, ^11^ we applied a two‐cutoff strategy (0.214 and 0.391 pg/mL), optimizing sensitivity and specificity at 95%. This approach achieved excellent accuracy, PPV, and NPV values, fully consistent with the minimal acceptable performance proposed by the Global CEO Initiative on Alzheimer's Disease,^12^ thus confirming the clinical applicability of plasma p‐tau217 as a diagnostic tool for AD.^12^ Examining the distribution of positive, negative, and indeterminate (“gray zone”) cases revealed that the proportion of gray zone results increased at earlier stages of cognitive decline. This emphasizes the need for an additional approach to resolve indeterminate p‐tau217 results, enabling a more precise biological diagnosis of AD.

A GLM demonstrated that p‐tau181 effectively helps to resolve uncertainty within the p‐tau217 gray zone, supporting its use as a secondary classifier. Specifically, we propose employing p‐tau181 as a dichotomous variable (positive/negative based on the validated cutoff) to refine patient classification as “likely AD” or “non‐AD.” The sequential model using p‐tau217 followed by p‐tau181 achieved excellent overall accuracy, PPV, and NPV, correctly reclassifying 77.4% of previously unclassified patients, thus being highly valuable in resolving indeterminate cases. This strategy confirms p‐tau217 as the best single biomarker for identifying AD pathology from the earliest stages, while p‐tau181 might be considered as a complementary biomarker which can further enhance classification precision within indeterminate cases.

Based on these results, we recommend the clinical use of plasma biomarkers, employing p‐tau217 as the primary diagnostic marker and p‐tau181 as a secondary tool to resolve gray zone cases. Indeed, our findings support the strong predictive value of plasma p‐tau217 for identifying underlying AD pathology. Moreover, p‐tau181 appears to be a promising complementary biomarker for clarifying indeterminate (“gray zone”) cases defined by p‐tau217 levels. Specifically, the availability of both measures may help refine classification, as p‐tau181 positivity among gray‐zone cases could indicate a higher likelihood of underlying AD pathology, whereas p‐tau181 negativity would support a non‐AD profile. In particular, considering the low risk of false negatives associated with plasma p‐tau217, a targeted diagnostic approach could be proposed: confirmatory CSF or amyloid‐PET assessments may be warranted only in patients within the p‐tau217 gray zone who also test positive for p‐tau181, while such procedures could reasonably be avoided in those who are p‐tau181 negative. In more details, we propose a flowchart for plasma biomarker interpretation:

- **Clearly negative p‐tau217** → AD pathology can be reasonably excluded.
- **Clearly positive p‐tau217** → AD pathology is highly suspected.
- **Gray zone p‐tau217** → test plasma p‐tau181:
  ‐ *p‐tau181 negative*: AD can be excluded with minimal risk of false negatives; consider retesting over time if symptoms progress.
  ‐ *p‐tau181 positive*: perform second‐level investigations (CSF or amyloid‐PET) for diagnostic confirmation.

This strategy could optimize resource use while maintaining diagnostic accuracy in the early stages of cognitive decline.

Our study has some limitations. First, renal function was not considered in these analyses, since only three patients had mild impaired renal function. Similarly, other systemic comorbidities that might influence plasma biomarker levels were not considered. Second, healthy controls were not included. Third, not all patients underwent both CSF biomarkers analysis and amyloid‐PET scans. Moreover, plasma biomarkers were analyzed using two different analytical platforms; therefore, possible inter‐platform variability cannot be definitively excluded. Furthermore, the limited number of gray‐zone patients may have reduced statistical power of the analyses, thus limiting interpretation and generalizability of the results. Finally, non‐AD demented patients were not included in this study, thus potentially introducing a bias when estimating BBMs’ accuracies.

Nevertheless, this study also has notable strengths. It focused not only on MCI but also on SCD, an even earlier stage of cognitive decline, which is a key target for early identification of individuals at risk of developing AD dementia. Furthermore, patients’ classification as carriers or noncarriers of AD was based on the new Revised Criteria, including the updated definition of Core1 biomarkers. Finally, to the best of our knowledge, this is the first that evaluated the use of other biomarkers to resolve p‐tau217 gray zone thus improving patients’ classification.

In conclusion, our findings confirm plasma p‐tau217 as the most accurate and reliable BBM for identifying AD across all clinical stages in real world context. Implementing a two cut offs approach allows for high diagnostic precision, meeting international performance standards, while the complementary use of p‐tau181 further improves classification in gray‐zone cases. Together, these results support the integration of plasma p‐tau217 and p‐tau181 into clinical diagnostic workflows as a minimally invasive, scalable, and cost‐effective strategy for the early biological detection of AD.

## CONFLICT OF INTEREST STATEMENT

The authors declare that they have no competing interests. Author disclosures are available in the Supporting Information.

## ETHICS STATEMENT

Study procedures and data analysis were performed in accordance with the Declaration of Helsinki and with the ethical standards of the Committee on Human Experimentation of our Institute. The study was approved by the local Institutional Review Board (reference 15691oss). All individuals involved in this research agreed to participate and agreed to have details and results of the research about them published.

## Supporting information



Supporting Information

Supporting Information

Supporting Information

Supporting Information

Supporting Information

## Data Availability

All study data, including raw and analyzed data, and materials that support the findings of this study are available from the corresponding author (B.N.) upon reasonable request.
