# Supplementary material for: Refining Alzheimer's disease biological diagnosis with plasma biomarkers: Resolving p‐tau217 “gray zone” with p‐tau181 integration
Source: Alzheimers Dement (Amst). 2026 Feb 15;18(1):e70285. doi: 10.1002/dad2.70285 (PMC12906650; doi:10.1002/dad2.70285)
Supplement: Supplementary file 1 — Supporting Information [file DAD2-18-e70285-s005.docx]

**Supplementary Materials**

**Collection of AD biomarker**

The CSF samples were collected by lumbar puncture, then immediately centrifuged and stored at −80 °C until performing the analysis. Aβ1–42, Aβ42/40 ratio, t-tau, and p-tau were measured using a chemiluminescent enzyme immunoassay (CLEIA) analyzer LUMIPULSE G600 (Lumipulse Beta Amyloid1–40, Lumipulse Beta Amyloid1–42, Lumipulse GTotal Tau, and Lumipulse GPhospho Tau (181)). Reagent kits were obtained from Fujirebio. Cut-off values for CSF biomarkers were determined following Fujirebio guidelines (diagnostic sensitivity and specificity using clinical diagnosis and the follow-up golden standard as of November 19th, 2018).

Amyloid PET imaging was performed according to national and international standards^1^, with any of the available fluorine18-labeled tracers (18Florbetaben [FBB]-Bayer-Pyramal, 18Flutemetamol [FMM]-General Electric). Images were rated as either positive or negative according to criteria defined by the manufacturers.

**Plasma biomarkers’ analyses**

Blood samples were collected by venipuncture into standard polypropylene EDTA test tubes (Sarstedt, Nümbrecht, Germany). Plasma was isolated from peripheral blood sample within 2 h of collection. Blood sample was centrifugated at 1300 rcf for 15 min. One mL plasma aliquots were pipetted into polypropylene cryotubes and stored at -80°C until tested. Plasma p-tau-217 levels were measured using fully automated CLEIA on the LUMIPULSE G600II system according to the manufacturer guidelines (Lumipulse® assay, Fujirebio, RUO for research use only).

Plasma p-tau181, NfL and GFAP analyses were performed on the automated Single molecule assay (Simoa) SR-X platform (Quanterix corp.). For GFAP and NfL measurement, the Simoa Human Neurology 2-Plex B assay (N2PB) (Item 103520) was used. The assay range of GFAP was 0 - ~40000 pg/mL, instead of NfL was 0 - ~2000 pg/mL. The N2PB kit analytical Lower Limit of Quantification (LLOQ) was for GFAP 4.15 pg/mL (pooled CV 16%, mean recovery 101%; Functional LLOQ=16.6 pg/mL) and for NfL 0.400 pg/mL (pooled CV 18%, mean recovery 101%; Functional LLOQ = 1.60 pg/mL). The N2PB kit Limit of Detection (LOD) was 0.410 pg/mL (range 0.133-0.740 pg/mL) for GFAP and 0.071 pg/mL (range 0.012-0.149 pg/mL) for NfL measurements. Quality controls (1= low protein level, 2= high protein level) were included in the run (GFAP control 1=94.8 pg/mL, control 2=12701 pg/mL; NfL control 1=4.21 pg/mL, control 2=556 pg/mL). Plasma p-tau181 was detected using the Simoa® pTau-181 Advantage V2.1 Kit (Item 104111). The Analytical LLOQ of the pTau 181 kit was 2.23 pg/mL (pooled CV 19.0%, mean recovery 97.0%; Functional LLOQ =8.92 pg/mL), instead the LOD was 1.04 pg/mL (range 0.146-1.750 pg/mL). pTau 181 quality controls were: control

1=50.5 pg/mL, control 2=530 pg/mL. The N2PB and pTau 181 kit analysis in all samples were performed in a single run basis, respectively. All plasma samples were diluted to 1:4. A reference calibration curve was established using serially diluted calibrators, provided by Quanterix. Plasma samples, calibrators, and controls were run in duplicate.

**Apolipoprotein E ε4 genotyping**

A standard automated method (QIAcube, QIAGEN) was used to isolate DNA from peripheral blood samples. *APOE* genotypes were investigated by high-resolution melting analysis (HRMA) ^2^. Two sets of PCR primers were designed to amplify the regions encompassing rs7412 [NC_000019.9:g[M13] [GG14] .45412079C>T] and rs429358 (NC_000019.9:g.45411941T>C). The samples with known *APOE* genotypes, which had been validated by DNA sequencing, were used as standard references. *APOE* genotype was coded as *APOE* ε4- (no *APOE* ε4 alleles) and *APOE* ε4+ (presence of one or two *APOE* ε4 alleles).

REFERENCES

1. Minoshima S, Drzezga AE, Barthel H, et al. SNMMI Procedure Standard/EANM Practice Guideline for Amyloid PET Imaging of the Brain 1.0. *J Nucl Med*. 2016;57(8):1316-1322. doi:10.2967/jnumed.116.174615

2. Sorbi S, Nacmias B, Forleo P, et al. ApoE allele frequencies in Italian sporadic and familial Alzheimer’s disease. *Neurosci Lett*. 1994;177(1-2):100-102. doi:10.1016/0304-3940(94)90054-x

**Supplementary Tables**

**Supplementary Table 1. Clinical and biological features of patients who underwent Amyloid PET as the only assessment of AD pathology**

|  | **Amyloid PET**  **(N=31)** |
| --- | --- |
| Age at plasma collection | 69.39±8.56 |
| Sex (F – M) | 21 -10 |
| *APOE* ε4 | 38.46% |
| MMSE | 25.93±4.76 |
| Clinical Diagnosis  SCD  MCI  AD-d |  |
|  | 14 (45.16%) |
|  | 11 (35.48%) |
|  | 6 (19.36%) |
| Positive Amyloid PET | 45.16% |

Values are reported as mean and standard deviation or percentage.

**Supplementary Table 2. Clinical and biological feature of patients who underwent CSF biomarkers analysis to assess AD pathology**

|  | **CSF analysis**  **(N=370)** |
| --- | --- |
| Age at plasma collection | 69.43±8.47 |
| Sex (F – M) | 218 - 152 |
| *APOE* ε4 | 42.77% |
| MMSE | 25.95±4.68 |
| Clinical Diagnosis  SCD  MCI  AD-d |  |
|  | 64 (17.30%) |
|  | 177 (47.84%) |
|  | 129 (34.86%) |
| Positive CSF Aβ42/40 ratio | 56.22% |
| Positive CSF p-tau181/Aβ42 ratio | 62.70% |
| Positive CSF Aβ42/40 and  p-tau181/Aβ42 ratios | 54.05% |

Values are reported as mean and standard deviation or percentage.

**Supplementary Table 3. Logistic regression analysis to predict AD pathology defined by the positivity of Core1 biomarkers**

|  | **B** | ***p*** | **OR** | **95% C.I.** | |
| --- | --- | --- | --- | --- | --- |
|  |  |  |  | **lower** | **upper** |
| Age at plasma collection | -0.04 | 0.396 | 0.95 | 0.84 | 1.06 |
| Plasma p-tau217 | 9.16 | **<0.001** | 9531.73 | 134.47 | 3604426 |
| Plasma p-tau181 | 0.68 | 0.085 | 1.98 | 0.95 | 4.70 |
| Plasma NfL | -0.02 | 0.647 | 0.98 | 0.89 | 1.07 |
| Plasma GFAP | 0.003 | 0.456 | 1.00 | 0.99 | 1.01 |
| *APOE* genotype | 0.96 | 0.194 | 2.63 | 0.61 | 11.92 |
| Sex | 0.52 | 0.482 | 1.69 | 0.40 | 7.87 |
| Clinical Diagnosis |  |  |  |  |  |
| MCI | 1.10 | 0.224 | 3.01 | 0.54 | 21.28 |
| AD-d | 18.42 | 0.991 | 99660730 | 0 | NA |

Regression Coefficients (B), p-value (p), Odds Ratio (OR) and 95% Confidence Intervals (95% C.I.) for covariates included in the regression models are reported. Significant differences at *p*<0.05, in **bold characters**.

**Supplementary Table 4. Metric comparison between plasma p-tau217 alone and the combined use of p-tau217 and p-tau181 in discriminating Core1 status.**

| **Metrics** | **Plasma p-tau217** | **Combined use of plasma**  **p-tau217 and p-tau181** | ***p*** |
| --- | --- | --- | --- |
| Accuracy | 94.30% (91.08–97.58) | 92.00% (88.46–95.54) | 0.336 |
| PPV | 95.58% (92.68–98.47) | 92.25% (88.74–95.76) | – |
| NPV | 92.59% (89.91–96.28) | 91.67% (88.06–95.28) | – |

The table shows the accuracy, positive predictive value (PPV), and negative predictive value (NPV) with their 95% confidence intervals (CI) of plasma p-tau217 alone and of the combined use of p-tau217 and p-tau181. *p* value from boostrap comparing each p-tau217 accuracy with that of the combined use.
