# Supplementary material for: Refining Alzheimer's disease biological diagnosis with plasma biomarkers: Resolving p‐tau217 “gray zone” with p‐tau181 integration
Source: Alzheimers Dement (Amst). 2026 Feb 15;18(1):e70285. doi: 10.1002/dad2.70285 (PMC12906650; doi:10.1002/dad2.70285)
Supplement: Supplementary file 2 — Supporting Information [file DAD2-18-e70285-s002.pdf]

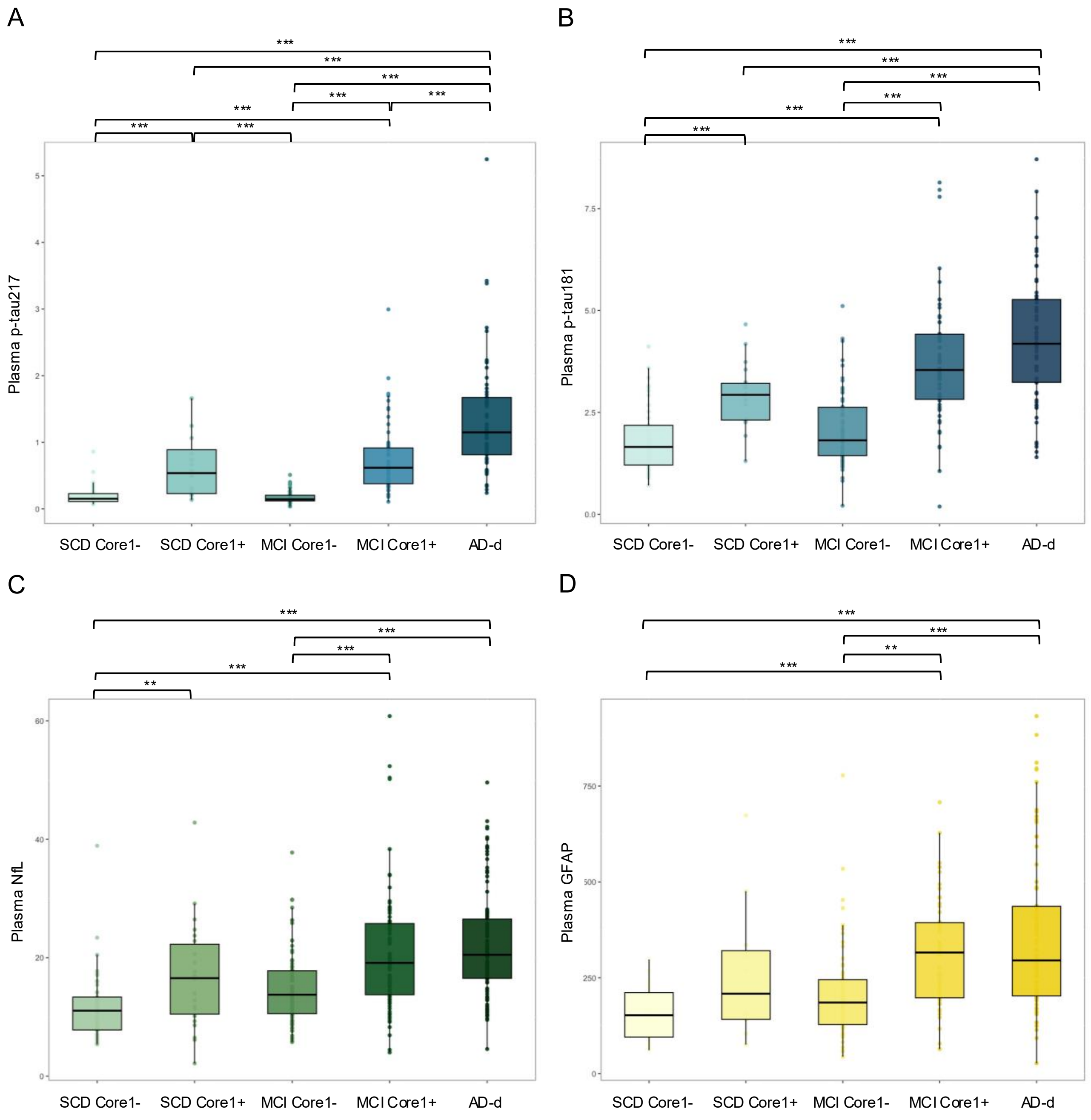

Supplementary Figure 1. Plasma biomarkers levels in Subjective Cognitive Decline (SCD), Mild Cognitive Impairment (MCI) and Alzheimer's Disease demented (AD-d) patients classified according to the Alzheimer's Association Revised Criteria for Alzheimer's Disease diagnosis.

Values quoted in the y-axis indicate plasma biomarkers levels (A. Plasma p-tau217; B. Plasma p-tau181; C. Plasma NfL; D. Plasma GFAP). Horizontal bars indicate significant differences between groups (SCD Core1-, SCD Core1+, MCI Core1-, MCI Core1+ and AD-d). * $p<0.05$; ** $p<0.01$; *** $p<0.001$.
