# Supplementary material for: Refining Alzheimer's disease biological diagnosis with plasma biomarkers: Resolving p‐tau217 “gray zone” with p‐tau181 integration
Source: Alzheimers Dement (Amst). 2026 Feb 15;18(1):e70285. doi: 10.1002/dad2.70285 (PMC12906650; doi:10.1002/dad2.70285)
Supplement: Supplementary file 3 — Supporting Information [file DAD2-18-e70285-s001.pdf]

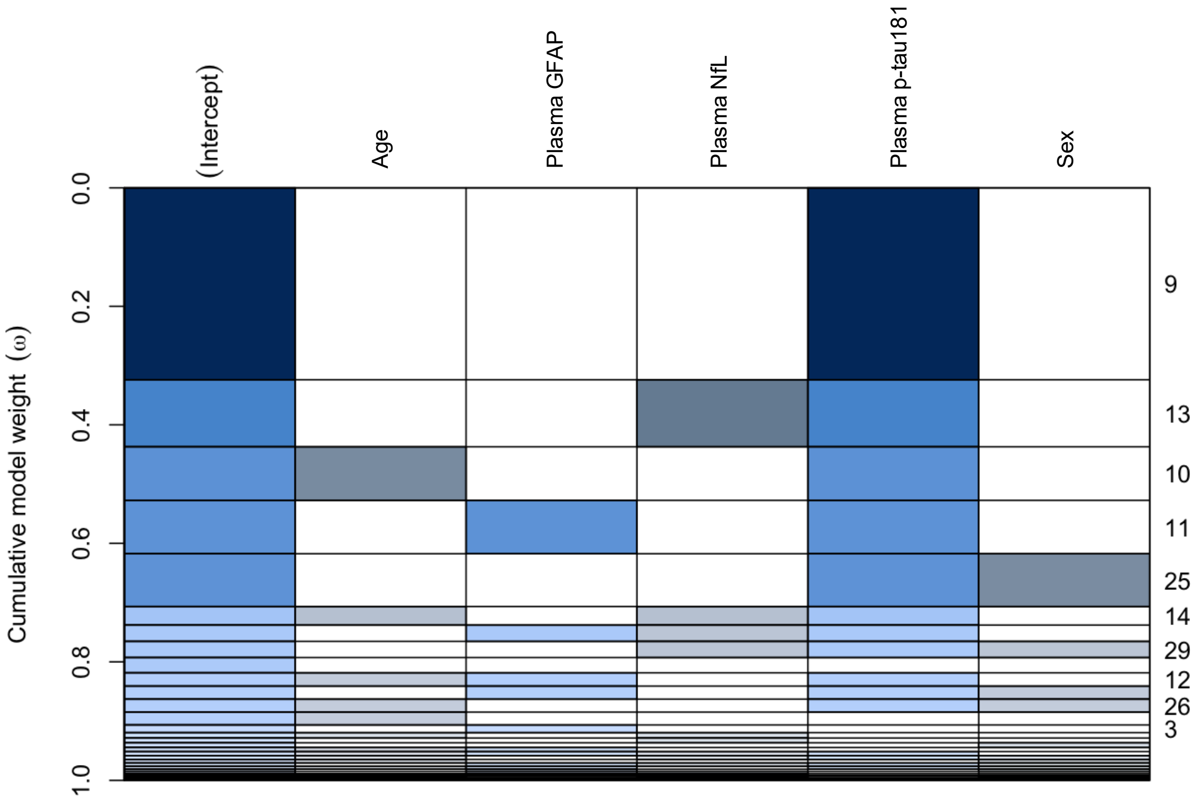

Supplementary Figure 2. Model selection for predicting Core1 positivity in patients in p-tau217 gray zone using plasma biomarkers and demographic covariates

Heatmap showing the top-ranked logistic regression models generated using AICc-based model selection (dredge). Columns represent predictors included in each model, and rows correspond to individual models ordered by cumulative model weight. Darker shading indicates inclusion of a predictor in a given model. The y-axis shows the cumulative weight (ω) of each model, reflecting its relative support among all candidate models. Plasma p-tau181 consistently appears in the highest-weight models, indicating it is the strongest predictor of Core1 positivity, whereas other biomarkers and covariates are included less frequently.
