# Supplementary material for: Refining Alzheimer's disease biological diagnosis with plasma biomarkers: Resolving p‐tau217 “gray zone” with p‐tau181 integration
Source: Alzheimers Dement (Amst). 2026 Feb 15;18(1):e70285. doi: 10.1002/dad2.70285 (PMC12906650; doi:10.1002/dad2.70285)
Supplement: Supplementary file 4 — Supporting Information [file DAD2-18-e70285-s004.pdf]

	Core1-	Core1+	
P-tau217 -	75	6	81
P-tau217 Gray Zone	21	22	43
P-tau217 +	5	108	113
	101	136	

Using p-tau181 in the gray zone

Available for 31 patients

	Core1-	Core1+	
P-tau181 -	13	2	15
P-tau181 +	5	11	16
	18	13	

	Core1-	Core1+	
Plasma -	88	8	96
Plasma +	10	119	129
	98	127	

Supplementary Figure 3. Improvement of Core1 classification by incorporating plasma p-tau181 in p-tau217 gray zone patients

Confusion matrices showing the classification of patients into Core1− and Core1+ based on plasma p-tau217 alone (top left), the subset of gray zone patients reclassified using plasma p-tau181 (top right), and the final classification after combining both biomarkers (bottom). Incorporation of p-tau181 enabled correct classification of patients initially unclassifiable with p-tau217 alone.
